# A platform for phenotyping disease progression and associated longitudinal risk factors in large-scale EHRs, with application to incident diabetes complications in the UK Biobank

**DOI:** 10.1093/jamiaopen/ooad006

**Published:** 2023-02-09

**Authors:** Do Hyun Kim, Aubrey Jensen, Kelly Jones, Sridharan Raghavan, Lawrence S Phillips, Adriana Hung, Yan V Sun, Gang Li, Peter Reaven, Hua Zhou, Jin J Zhou

**Affiliations:** Department of Biostatistics, University of California, Los Angeles, California, USA; Department of Biostatistics, University of California, Los Angeles, California, USA; Department of Computer Science, Columbia University, New York, New York, USA; Division of Hospital Medicine, University of Colorado School of Medicine, Aurora, Colorado, USA; Rocky Mountain Regional VA Medical Center, Aurora, Colorado, USA; Division of Endocrinology, Emory University School of Medicine, Atlanta, Georgia, USA; Atlanta VA Medical Center, Decatur, Georgia, USA; VA Tennessee Valley Healthcare System, Nashville, Tennessee, USA; Vanderbilt University, Nashville, Tennessee, USA; Department of Epidemiology, Emory University, Atlanta, Georgia, USA; Department of Biostatistics, University of California, Los Angeles, California, USA; Department of Computational Medicine, David Geffen School of Medicine, University of California, Los Angeles, California, USA; Phoenix VA Health Care System, Phoenix, Arizona, USA; Department of Biostatistics, University of California, Los Angeles, California, USA; Department of Computational Medicine, David Geffen School of Medicine, University of California, Los Angeles, California, USA; Department of Biostatistics, University of California, Los Angeles, California, USA; Phoenix VA Health Care System, Phoenix, Arizona, USA; Department of Medicine, David Geffen School of Medicine, University of California, Los Angeles, California, USA

**Keywords:** phenotyping, diabetes, diabetes complications, disease progression, electronic health records, time-to-event

## Abstract

**Objective:**

Modern healthcare data reflect massive multi-level and multi-scale information collected over many years. The majority of the existing phenotyping algorithms use case–control definitions of disease. This paper aims to study the time to disease onset and progression and identify the time-varying risk factors that drive them.

**Materials and Methods:**

We developed an algorithmic approach to phenotyping the incidence of diseases by consolidating data sources from the UK Biobank (UKB), including primary care electronic health records (EHRs). We focused on defining events, event dates, and their censoring time, including relevant terms and existing phenotypes, excluding generic, rare, or semantically distant terms, forward-mapping terminology terms, and expert review. We applied our approach to phenotyping diabetes complications, including a composite cardiovascular disease (CVD) outcome, diabetic kidney disease (DKD), and diabetic retinopathy (DR), in the UKB study.

**Results:**

We identified 49 049 participants with diabetes. Among them, 1023 had type 1 diabetes (T1D), and 40 193 had type 2 diabetes (T2D). A total of 23 833 diabetes subjects had linked primary care records. There were 3237, 3113, and 4922 patients with CVD, DKD, and DR events, respectively. The risk prediction performance for each outcome was assessed, and our results are consistent with the prediction area under the ROC (receiver operating characteristic) curve (AUC) of standard risk prediction models using cohort studies.

**Discussion and Conclusion:**

Our publicly available pipeline and platform enable streamlined curation of incidence events, identification of time-varying risk factors underlying disease progression, and the definition of a relevant cohort for time-to-event analyses. These important steps need to be considered simultaneously to study disease progression.

## INTRODUCTION

The paradigm of precision medicine has expanded the use of very large, interoperable, and longitudinal cohorts. A key advantage of many healthcare related datasets, whether primary care records, claims data, or registry data,^1^ is their accumulation of patient information over long periods of time.^2^ They offer an excellent resource to longitudinally monitor clinical biomarkers whose fluctuations might influence the progression of diseases. Rich and time-stamped information stored in electronic health records (EHRs) make more accurate and standardized phenotyping possible.^3^^,^^4^ Indeed, temporal sequential data representations mined from EHRs have been demonstrated to offer a more accurate phenotype classification than its individual components.^4^^,^^5^ However, these studies modeled diseases as discrete events (eg, cases vs controls). Deep time-to-event phenotyping requires more sophisticated analytics beyond the case–control classification, and the incorporation of domain knowledge remains critical.^2^^,^^3^^,^^6^^,^^7^ Our objective is to incorporate time to phenotype disease progression, use diabetes mellitus (DM) and its vascular complication as an example, and provide an associated cohort definition for time-to-event analysis in the UK Biobank (UKB) study.^8^^,^^9^

DM is a progressive disease associated with multiple risk factors, such as hyperglycemia and elevated blood pressure. These risk factors help drive the incidence of complications, including cardiovascular disease (CVD), diabetic kidney disease (DKD), diabetic retinopathy (DR), and neuropathy.^10–12^ While a large number of studies examined factors associated with the prevalence, or incidence, of diabetes, fewer studies have used biobank-scale health care datasets to examine the development of diabetes-related complications.^13^ A notable reason stems from the lack of a uniform phenotyping definition of diabetes and diabetes-related complications in EHRs. UKB is one of the largest biobanks globally, with over 500 000 participants. The UKB continues to enhance links between their information and the UK primary care EHR data.^8^^,^^9^ However, researchers working with UKB-linked EHR data face significant challenges, as these EHR systems are designed to collect patient information for administration and management purposes, not for analysis and research. For example, UKB EHR data are an amalgamation of different sources, recorded using different methods (containing more than 500 000 terms to record information). Our current contribution is to provide tools for quantifying disease incidence and progression, in addition to relevant longitudinal biomarkers, thus enabling more sophisticated time-to-event analysis.

We established diabetes and diabetes complication diagnoses by systematically consolidating disparate sources of clinical data from patient questionnaires, hospital records, death records, and primary care data released by UKB. We focus on cardiovascular complications reflecting ischemic events and microvascular complications, including DKD and DR. Furthermore, we phenotyped longitudinal risk factors for the aforementioned complications. We documented our phenotyping framework using an R package, bookdown, and have made it publicly available as a short book, including code lists, procedures, and implementations (https://dohyunkim116.github.io/ukbiobank-phenotyping-book/). To demonstrate the utility of our phenotyping framework, we assess its ability to reproduce known associations of risk factors with DM complications using a prospective design and Cox proportional hazards models. In addition, we build several DM complication prediction engines. Although this paper focuses on UKB data, some of the controlled clinical terminologies used in UK EHR are applicable to US data sources. Therefore, our work can benefit other large-scale data resources such as Electronic Medical Records and Genomics (eMERGE),^14^ BioVU,^15^ Million Veteran Program,^16^ and All Of Us.^17^

## MATERIALS AND METHODS

### The UKB data resources

The UKB is a prospective cohort study with deep genetic and phenotypic data. Record linkage to Health Episode Statistics (England), Patient Episode Database for Wales, and the Scottish Morbidity Records (Scotland) was used to identify the date and cause of hospital admissions. Hospital admission records were available until February 2018 for the full UKB cohort (noted as “UKB data”), whereas linkage to primary care records was available for 45% of the UKB cohort until the end of 2017 (noted as “UKB Primary Care data”). Each record has an entry for a clinical term under the format of either Read v2 or Read v3/CTV3. Although Read v2 or Read v3/CTV3 are the primary care healthcare concepts used in the UK, Read v3/CTV3 is one of the core components of SNOMED-CT, an international standard for recording information across healthcare settings and a suite of designated standards for use in the United States.

### Outcome definitions

**Figure 1.**
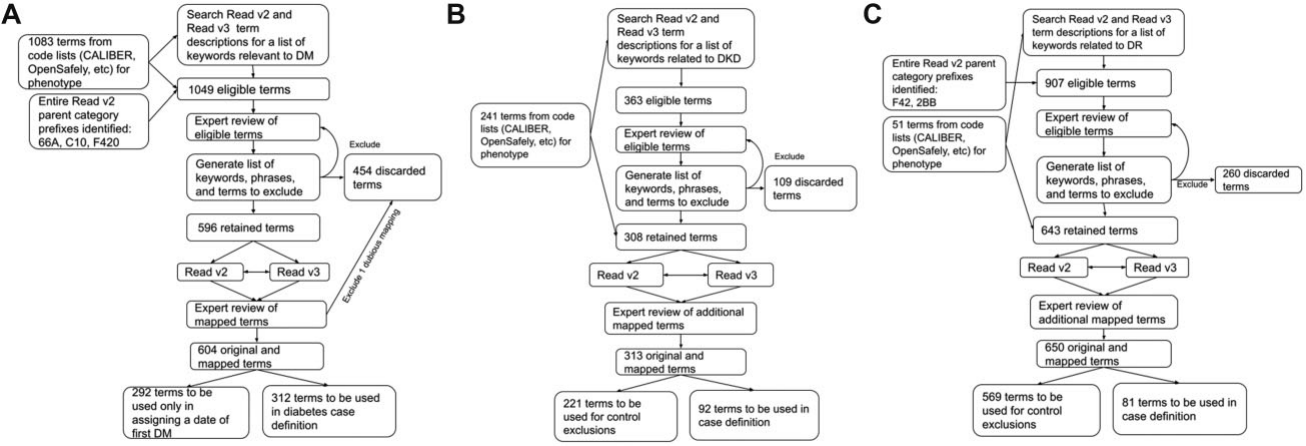
Flow chart to identify candidate terms for phenotyping (A) diabetes, (B) diabetic kidney disease (DKD), and (C) diabetic retinopathy (DR). CALIBER: cardiovascular disease research using linked bespoke studies and electronic health records, https://www.ucl.ac.uk/health-informatics/research/caliber; Read v3 (CTV3): Clinical Terms Version 3; OpenSAFELY: a secure, transparent, open-source software platform for analysis of electronic health records.

We ascertained DM and three primary DM macro- and microvascular complications: a composite CVD outcome (reflecting myocardial infarction [MI], unstable angina [UA], ischemic stroke [IS], and percutaneous coronary intervention [PCI]), DKD, and DR. For UKB data, the outcome definitions were defined through sets of predefined UKB fields mapping to different outcomes. The UKB fields belonged to different classes of the fields, including UKB First Occurrence fields, algorithmically defined fields, fields containing ICD-10 codes, OPCS4 codes, self-reported illness codes, self-reported operation codes and custom defined fields (Supplementary Table S1). For UKB Primary Care data, the outcome definitions (for DM, DKD, and DR only) were defined through code lists from CALIBER and OpenCodelists mapping to different outcomes. We further refined a list of terms defining the outcomes by manual inspection and inputs from experts. Figure 1 details procedures identifying and refining candidate terms. A full list of codes and data fields used to define a specific outcome is shown in Supplementary Table S2 and Supplementary Text. We obtained the most up-to-date information by using clinical terms directly from hospital admission, death, and primary care records, even if the term was already captured by a First Occurrence field. DM was defined and categorized into one of three types, Type 1, Type 2, and Uncertain. Detailed descriptions of each condition can be found in the Supplementary Text.

### Phenotyping incidence events and cohort definition

#### Procedures

**Figure 2.**
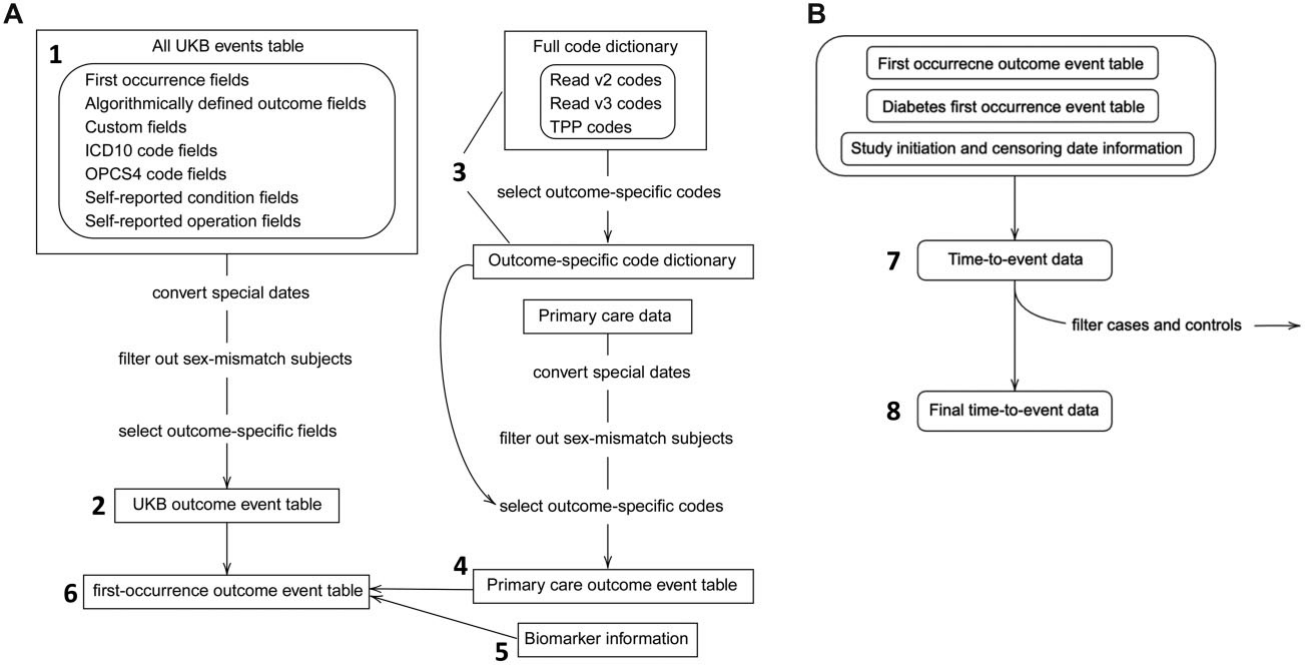
(A) Flowchart of outcome event table generation steps. UK Biobank (UKB) outcome event tables were generated by searching relevant outcome fields from all UKB event tables. UKB event table combines all clinical event fields available from UKB assessment center data. Primary care outcome event tables were generated by searching the outcome-specific codes from the primary care data. The outcome-specific code dictionary was generated by searching the full dictionary for relevant codes and descriptions. The full dictionary is a combination of Read v2, Read v3, and TPP codes. We combined information from UKB outcome event table, primary care outcome event table, and biomarker information to create a first-occurrence outcome event for each outcome. Numbers in labels correspond to the steps in the Phenotyping procedure section. (B) Flowchart of time-to-event data generation steps. Time-to-event data for an outcome required first occurrence outcome event table, diabetes first-occurrence event table and a dataset containing subjects’ study initiation dates (ie, index date) and censoring dates. Time-to-event data were subject to filtering based on certain exclusion criteria for an outcome. ICD10: 10th revision of the International Statistical Classification of Diseases; Read v3 (CTV3): Clinical Terms Version 3; OPCS4: Office of Population Censuses and Surveys (OPCS) Classification of Interventions and Procedures version 4; TPP codes: TPP (https://tpp-uk.com/) is a data system supplier in UK and has their code lists. A list of TPP local codes that are present in the current extract and their definitions can be found in Data Showcase Encoding 8708.

We used the following steps to phenotype DM and DM complications (Figure 2).

1. *Generate a master event table.* To capture events related to an outcome from the “UKB data,” we first created a master event table containing all available clinical event fields and associated event dates,
  - first-occurrence outcome fields,
  - algorithmically defined outcome fields,
  - code event fields: ICD-10, OPCS4, self-reported condition, and self-reported operation codes, and
  - custom fields (used for phenotyping of DR events).
2. *Generate UKB outcome event table.* Using the fields identified in Supplementary Table S2, we searched the master event table to generate a UKB event table that includes all events related to each outcome.
3. *Generate master primary care code dictionary and outcome-specific code dictionary.* To identify events related to an outcome from the “UKB Primary Care data,” we created a master primary care code dictionary that combines Read v2, Read v3/CTV3, and TPP Local term dictionaries. Using defined codes and descriptions for an outcome, we searched the master code dictionary to generate outcome-specific code dictionaries.
4. *Generate primary care outcome event tables.* Using the outcome-specific dictionaries, we searched the “UKB Primary Care data” to generate primary care outcome event tables, which include all events related to each outcome.
5. *Generate biomarker trajectory data.* We extracted biomarker measurements of subjects using the “UKB primary care data” and the “UKB data.” We created trajectory data for biomarkers, including glucose, HbA1c, urine albumin, urine creatinine, urine albumin-to-creatinine ratio (uACR), serum creatinine, blood pressure, total cholesterol (TC), high-density lipoproteins (HDL), low-density lipoproteins (LDL), and triglycerides. The terms and fields for these biomarkers can be found in Supplementary Table S3. Using the curated trajectory data, we created event tables capturing the occurrence of macroalbuminuria, microalbuminuria, and prolonged low estimated glomerular filtration rate (eGFR) events. These event tables were used to capture DKD events and refine the time-to-event table for DKD. We further elaborate on the biomarker extraction step in the subsection *Covariate and Biomarker Extraction*.
6. *Generate outcome event table.* The event tables for DM, CVD, DKD, and DR were created by merging the UKB outcome event table generated in (2) and the primary care outcome event table generated in (4). The first-occurrence event tables and risk set exclusion event tables (Supplementary Table S2**)** associated with certain outcomes were also created.
7. *Generate an initial time-to-event table*. Time-to-event tables for CVD, DKD, and DR were created by merging the first occurrence DM event table, complication event tables and a demographics table which included censoring dates. An event status was positive if a diabetes subject had an incidence of a certain complication outcome before the censoring date; otherwise, a subject was at risk. A subject’s censoring date was defined as the earliest date among loss-to-follow-up date, showcase censoring date (as indicated by reduced EHR data availability) and the date of death (for a deceased participant). The fields used to determine the censoring dates are described in Supplementary Table S4.
8. *Generate refined time-to-event table.* Separately for each complication, we excluded participants from the time-to-event table if a complication event occurred before the first documented evidence of diabetes or after the censoring date. For each outcome, we also used an associated exclusion event table generated in (5) to exclude additional subjects in this risk set.

#### Additional exclusion considerations

Primary care data were only available for 45% of UKB participants. For DKD and DR, we required subjects in the risk set to be represented in the primary care data, unless they had an outcome event documented in hospital admissions or the death record. This is because a large proportion of DKD and DR events were ascertained using the primary care data and biomarker information, so we were not confident that participants without linked primary care data did not have complications. To preferentially capture diabetes-related kidney disease as opposed to kidney diseases arising from a different etiology, we required patients with events to have at least 5 years between the first evidence of diabetes and the complication occurrence. We also required patients in the risk set to have at least 5 years of follow-up time since the first evidence of diabetes. Other exclusion of conditions from the risk set was documented in Supplementary Table S2.

#### A prospective study design and cohort definition

**Figure 3.**
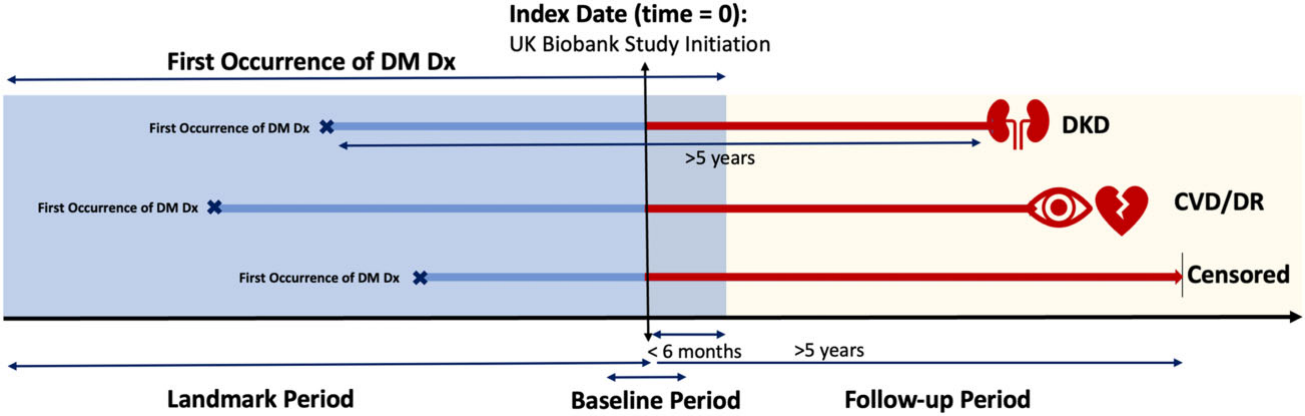
A prospective study design and landmark analysis. The index date is defined as the UKB study initiation date. The blue-shaded area is the landmark period, that is, from the first date of participants' primary care records to 6 months after the index date. We extract time-invariant and time-varying longitudinal measures of biomarkers from this period. In our study design, the first occurrence of DM Dx is required to be in the landmark period. The yellow-shaded area represents the follow-up period from the index date. We require the incidence of DM complications (ie, DKD, CVD, and DR) to be in this period. The baseline period up-to-6 months after the index date, during which participants' information was collected through UKB’s assessment center. To preferentially capture diabetes-related kidney disease as opposed to kidney diseases arising from a different etiology, we required patients with events to have at least 5 years between the first evidence of diabetes and the complication occurrence. We also required patients in the risk set to have at least 5 years of follow-up time since the first evidence of diabetes. CVD: cardiovascular disease; DKD: diabetic kidney disease; DR: diabetic retinopathy; DM: diabetes mellitus; Dx: diagnosis; X: event happened. | represents a censored event.

Using the developed phenotyping algorithm (Figure 2), we used a prospective study design to assemble the cohort and estimate the risks of known “risk” factors for DM complications (Figure 3). We define the index date (time 0) as the UKB study initiation date and follow the participants for the onset of macrovascular and microvascular conditions. One alternative index date could be the date of DM onset. In this manuscript, we assume the date of the first evidence of DM from EHR is the true date of DM onset. However, one may consider this date of DM onset unknown, which leads to different censoring mechanisms for complication events, for example, interval censoring. We briefly discuss this point in Supplementary Text. The time-to-event outcome for patients who had events was defined as the time between their index date and the date of their first recorded outcome event. The time-to-event outcome for patients in the risk set was defined as the time between the index date and the censoring date. Participants who had the event were excluded if the index date was not between the first evidence of diabetes and the date of complication event, while participants who did not have the event were excluded if the first evidence of diabetes was not before or within 6 months after the index date.

### Covariate and biomarker extraction

Patients’ race/ethnicity, year and month of birth, sex, body mass index (BMI), smoking status, and use of insulin, blood pressure lowering medication, or lipid-lowering medication were extracted from surveys collected at a UKB assessment center. Educational attainment was defined as the individual’s highest qualification, translated to the International Standard Classification of Education (ISCED) using the mappings in Ge et al.^18^ Physical activity was measured using weekly Total Metabolic Equivalent Task (MET) minutes. The Charlson Comorbidity Index (CCI) was generated using participants’ hospital admission records preceding their initiation into the study.^19^^,^^20^ To demonstrate genetic data usage in conjunction with our curated phenotypes, type 2 diabetes (T2D) polygenic risk scores (PRS) were generated for all individuals of European ancestry using the prepruned variants and weights from Mahajan et al,^21^ while type 1 diabetes (T1D) PRS were generated for the same individuals using the procedures from Sharp et al.^22^

We used two sources of information to extract biomarkers: measures from samples collected at participants’ visits to a UKB assessment center, and from the primary care database. To extract the latter, we reviewed clinical terms in UKB Resource *592*. We used a keyword-based search to identify relevant terms, and verified the code lists with the list prepared by Denaxas et al.^23^ The eGFR measurements were estimated from serum creatinine using the Chronic Kidney Disease Epidemiology Collaboration (CKD-EPI creatinine) equation.

### Statistical analysis

A landmark method was used to ascertain the known risk factors prior to the index date, including glycemic measures, blood pressures, and lipid measures. Specifically, we computed averages and estimates of variability of these biomarker measurements. We imputed the summary statistics of biomarker (eg, CV) measurements to obtain a more complete dataset for the primary analysis. Other imputed baseline covariates included sex, self-reported ethnicity, age at initiation date, smoking status, BMI, self-reported medication status for insulin, blood pressure drugs, and cholesterol drugs, CCI, ISCED level (greater than level 2 or not), and MET. We used the “UKB data” field number *21000* to broadly recategorize self-reported ethnicity into four groups: Asian, Black, Other, and White (Supplementary Table S5). Each variable was imputed using either predictive mean matching or model prediction procedure based on the variable’s distribution. A predictive mean matching procedure was adopted if the variable was categorical, integer-valued or if its distribution was skewed, as recommended by miceRanger R package.^24^ Otherwise, we used the model prediction procedure. Five imputed datasets were generated.

For our primary analysis, we implemented a pooled step-wise variable selection method^25^ for the Cox proportional hazards model to simultaneously analyze imputed data and select important variables contributing to the incidence of diabetes complications. Our base model includes sex and age, which were not subject to variable selection. Additional covariates were then added, including smoking status, BMI, self-reported medication status (insulin, blood pressure, and cholesterol drug), CCI, ISCED level, MET, PRS (type 1 and type 2 in European ancestry), and summary statistics of biomarker trajectories. Sensitivity analysis was conducted among the T2D cohort. We report hazard ratios with 95% confidence intervals. All analyses were performed using R version 4.0.2.

Furthermore, we conducted a risk prediction analysis. Using five imputed datasets, we split the data into training and validation sets in a 2 to 1 ratio, performed pooled step-wise variable selection on the training set, and computed risk scores using the validation set for each imputed dataset. Average risk scores (computed across imputed datasets) were used to assess the prognostic performances measured by the area under the receiver operating characteristic curve (AUC). The Kaplan–Meier (KM) curves comparing high- and low-risk groups with respect to median risk scores were also created for multivariable pooled step analysis. Finally, we sought to use non-European participants as an external validation cohort to evaluate prediction performance (PRS scores were not included).

## RESULTS

Phenotyping DM, DKD, and DR required the use of primary care code lists. We initially identified 1083 terms related to diabetes (Figure 1A) from CALIBER and OpenCodelists resources. Additional diabetes-related keywords were searched among Read v2 and Read v3/CTV3 dictionaries, and additional potential terms were gathered by including all “child codes” of several Read v2 “parent codes”. After excluding terms that did not occur at all in the “UKB Primary Care data,” 1049 eligible terms remained. Of these, 596 terms were retained after 454 terms and keywords were excluded by expert review because they lacked relevance to a diabetes diagnosis. We obtained 8 additional terms that mapped directly to an included term (either Read v2 to Read v3/CTV3, or vice versa), for a total of 604 terms. We split terms into two categories: (1) 312 codes sufficient to classify patients as having DM, and (2) 292 codes which were used only in the assignment of the first incidence date of the diabetes phenotype, for those patients who had codes from the first category. For DKD, 363 eligible terms were reviewed, of which 313 were retained (Figure 1B). Ninety-two of these terms were used to define the incident event of DKD, while 221 were used to exclude individuals from the risk set of DKD. For DR, 907 eligible terms were reviewed, of which 650 were retained. Eighty-one of these terms were used to define incident events of DR, while 569 were used to exclude individuals from the risk set of DR (Figure 1C).

### Cohort characteristics

**Figure 4.**
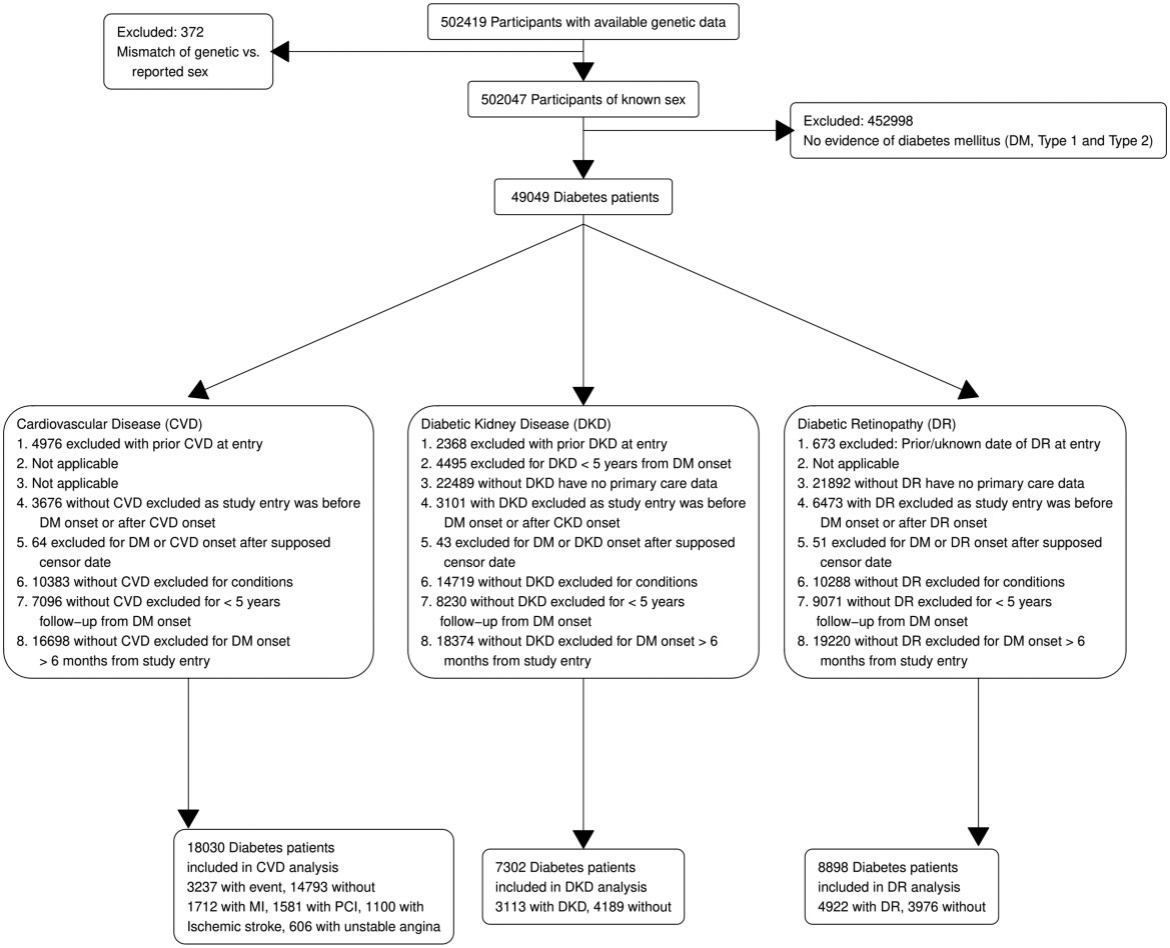
Flowchart of cohort curation for diabetes and diabetes complications. CVD: cardiovascular disease; DKD: diabetic kidney disease; DR: diabetic retinopathy; DM: diabetes mellitus; PCI: percutaneous coronary intervention.

Using the “UKB data” and the “primary care data,” we identified a total of 49 049 diabetes participants (Figure 4) who were categorized into either T1D, T2D, or “uncertain” (Supplementary Text). Among them, 1023 were T1D patients, and 40 193 were T2D patients. A total of 23 833 diabetes subjects had linked primary care records. Among them, 428 were T1D patients and 20 181 were T2D (Table 1).

**Table 1.** Cohort characteristics

	CVD cohort^a^				DKD cohort^b^				DR cohort^c^			
	Overall	No CVD	CVD		Overall	No DKD	DKD		Overall	No DR	DR	
	*n* = 18 030	*n* = 14 793	*n* = 3237	*P* ^c^	*n* = 7302	*n* = 4189	*n* = 3113	*P* ^c^	*n* = 8898	*n* = 3976	*n* = 4922	*P* ^c^
Ethnicity (%)				.003				<.001				.012
White	87	86	88		89	88	89		89	90	89	
Asian	7	7	7		7	7	6		7	6	7	
Black	4	4	3		3	2	3		2	2	3	
Other	2	3	2		2	2	2		2	2	2	
European (%)	61	61	60	.130	63	63	62	.494	63	64	62	.089
Primary care subjects (%)	48	49	46	.009	78	100	49	<.001	83	100	70	<.001
Sex (% males)	59	57	71	<.001	63	64	63	.350	61	60	62	.167
Age (years)	61 (54, 65)	60 (54, 65)	63 (58, 67)	<.001	61 (55, 66)	59 (53, 64)	64 (60, 67)	<.001	61 (55, 65)	60 (54, 65)	62 (56, 66)	<.001
BMI males (kg/m^2^)	30 (27, 33)	30 (27, 33)	31 (28, 34)	<.001	30 (27, 34)	30 (27, 33)	31 (28, 35)	<.001	30 (27, 34)	30 (28, 34)	30 (27, 34)	.741
BMI females (kg/m^2^)	31 (27, 36)	31 (27, 36)	32 (28, 37)	<.001	32 (28, 37)	31 (27, 36)	33 (29, 38)	<.001	32 (28, 37)	32 (27, 36)	32 (28, 37)	.027
Ever smoked (%)	50	48	58	<.001	54	51	59	<.001	54	53	55	.092
Smoking pack years	24 (14, 39)	23 (13, 36)	32 (18, 48)	<.001	27 (15, 42)	24 (14, 38)	31 (18, 48)	<.001	27 (16, 42)	26 (15, 40)	28 (16, 44)	.030
CCI (%)				<.001				<.001				<.001
0	63	66	47		53	65	37		57	62	53	
1	24	23	28		24	21	29		24	21	27	
2	8	7	14		12	9	16		10	9	11	
3	3	3	6		6	4	9		5	4	5	
4	1	1	3		2	1	5		2	2	2	
≥5	1	1	3		2	1	4		2	2	2	
MET (h/week)	23 (10, 51)	24 (10, 51)	21 (8, 49)	<.001	23 (9, 51)	25 (10, 54)	19 (7, 45)	<.001	23 (9, 51)	23 (9, 52)	22 (8, 50)	.113
ISCED level >2 (%)	59	61	52	<.001	56	61	48	<.001	57	58	57	.401
DM type (%)				<.001				<.001				<.001
Type 1	4	4	2		3	4	1		2	2	3	
Type 2	74	74	73		76	79	73		80	83	78	
Uncertain	23	22	25		21	17	26		18	15	19	
Insulin (%)	20	18	27	<.001	22	15	30	<.001	18	9.3	26	<.001
BP medication (%)	60	57	71	<.001	62	50	79	<.001	62	57	66	<.001
Cholesterol medication (%)	71	70	76	<.001	74	66	83	<.001	73	67	78	<.001
T1D PRS (tertile)				.744				.341				.348
1	30	30	31		31	31	32		31	31	32	
2	32	32	31		32	33	31		33	34	32	
3	38	38	38		37	36	37		36	35	36	
T2D PRS (tertile)				.075				.007				.002
1	18	18	18		18	19	17		17	18	16	
2	30	31	28		31	32	29		32	32	31	
3	52	51	54		51	49	54		51	49	53	
SBP (mmHg)	139 (130, 148)	138 (130, 148)	142 (132, 152)	<.001	138 (129, 147)	136 (128, 145)	140 (131, 150)	<.001	138 (130, 147)	138 (130, 146)	139 (130, 147)	<.001
DBP (mmHg)	82 (76, 87)	82 (77, 87)	81 (75, 87)	<.001	81 (76, 86)	82 (77, 86)	80 (74, 86)	<.001	82 (77, 86)	82 (77, 87)	81 (76, 86)	<.001
Chol (mmol/l)	4.6 (4.0, 5.3)	4.6 (4.0, 5.3)	4.6 (4.0, 5.2)	.014	4.6 (4.1, 5.2)	4.8 (4.2, 5.4)	4.4 (3.8, 5.0)	<.001	4.7 (4.1, 5.3)	4.8 (4.3, 5.5)	4.6 (4.0, 5.2)	<.001
Trig (mmol/l)	1.8 (1.3, 2.5)	1.8 (1.2, 2.5)	1.9 (1.3, 2.7)	<.001	1.8 (1.3, 2.6)	1.7 (1.3, 2.5)	2.0 (1.4, 2.8)	<.001	1.9 (1.3, 2.6)	1.9 (1.3, 2.6)	1.9 (1.3, 2.6)	.774
HDLc (mmol/l)	1.2 (1.0, 1.4)	1.2 (1.0, 1.4)	1.1 (1.0, 1.3)	<.001	1.2 (1.0, 1.4)	1.2 (1.0, 1.4)	1.1 (1.0, 1.3)	<.001	1.2 (1.0, 1.4)	1.2 (1.0, 1.4)	1.2 (1.0, 1.4)	.001
LDLc (mmol/l)	2.7 (2.2, 3.2)	2.7 (2.2, 3.2)	2.6 (2.2, 3.1)	.009	2.6 (2.2, 3.2)	2.7 (2.3, 3.3)	2.5 (2.1, 3.0)	<.001	2.7 (2.2, 3.2)	2.8 (2.3, 3.3)	2.6 (2.1, 3.1)	<.001
HbA1c (mmol/mol)	51 (44, 61)	51 (44, 60)	54 (46, 65)	<.001	52 (45, 62)	51 (43, 60)	54 (47, 64)	<.001	52 (44, 61)	49 (42, 57)	55 (47, 64)	<.001
Glucose (mmol/l)	7.0 (5.6, 9.4)	6.9 (5.5, 9.2)	7.4 (5.7, 10.1)	<.001	7.1 (5.7, 9.5)	6.9 (5.6, 9.1)	7.5 (5.8, 10.1)	<.001	7.2 (5.8, 9.5)	6.7 (5.5, 8.6)	7.7 (6.1, 10.2)	<.001
eGFR (ml/min/1.73 m^2^)	87.8 (75.5, 96.9)	88.4 (76.5, 97.4)	84.3 (70.7, 94.4)	<.001	83.4 (74.1, 93.1)	87.4 (79.8, 95.7)	75.3 (66.0, 86.8)	<.001	83.0 (72.3, 93.5)	82.9 (73.0, 93.3)	83.1 (71.5, 93.8)	.730
loguACR (log[g/mmol])	0.2 (−0.4, 0.9)	0.1 (−0.4, 0.8)	0.5 (−0.2, 1.5)	<.001	0.0 (−0.5, 0.7)	−0.3 (−0.7, 0.1)	0.7 (−0.1, 2.0)	<.001	0.2 (−0.4, 0.9)	0.1 (−0.5, 0.7)	0.2 (−0.4, 1.0)	<.001
Incidence rate^2^	16.31 (15.76–16.88)				46.22 (44.61–47.88)				71.79 (69.8–73.83)			

CVD: cardiovascular disease; DKD: diabetic kidney disease; DR: diabetic retinopathy; eGFR: estimated glomerular filtration rate; ISCED: International Standard Classification of Education, dichotomous variable, 1 if ISCED level was greater than 2 and 0 otherwise; Ever smoked, dichotomous variable, 1 if a subject has ever smoked and 0 otherwise; MET: metabolic equivalents to resting state (h/week); BMI: body mass index (kg/m^2^); CCI: Charlson Comorbidity Index; Chol: total cholesterol (mmol/l); Trig: triglycerides (mmol/l); HDLc: high-density lipoprotein cholesterol (mmol/l); LDLc: low-density lipoprotein cholesterol (mmol/dl); SBP: systolic blood pressure (mm Hg); DBP, diastolic blood pressure (mm Hg); eGFR: estimated glomerular filtration rate (ml/min/1.73 m^2^); uACR: urine albumin to creatinine ratio (g/mmol).

a Median (IQR) or percent or incidence rate (95% confidence interval);

b Per 1000 persons per year;

c Pearson's Chi-squared test or Wilcoxon rank sum test.

The incidence rates for CVD, DKD, and DR in our cohort were estimated to be 16.3, 46.2, and 71.8 in 1000-person-years, respectively. There were 18 030 diabetes patients (74% T2D patients) in the CVD cohort and 3237 of them were identified to have developed CVD events after the diagnosis of diabetes. Among diabetes patients, 7302 patients (76% T2D patients) were in the DKD cohort and 3113 of them had DKD events. There were 8898 diabetes patients (80% T2D patients) in the DR cohort and 4922 of them developed DR.

The median age at study initiation was 61 across all cohorts. Diabetes patients who developed CVD were generally older than those who did not (63 vs 60 years old), more likely to be an ever smoker (58% vs 48%), fewer hours of exercise (21 vs 24 h/week), higher baseline SBP, HbA1c, glucose, triglycerides, and uACR measures. Note that many biomarkers (eg, cholesterol, HDL, and LDL) were within normal or close to normal range, indicating the UKB cohort represents a relatively healthy population.^26^ Interestingly, T2D PRS tertiles are significantly associated with DKD and DR but not CVD. In the CVD cohort, 27% versus 18% of the patients who did versus did not develop CVD events reported insulin usage; while in DKD and DR cohorts, the proportions were 30% versus 15% and 26% versus 9.3%, respectively. In general, participants with DM complications tend to have higher proportions of medication usage. Among three conditions, DKD patients have the highest insulin, blood pressure, and cholesterol medication usage. Results are consistent with the literature that DKD patients have more comorbidities.^13^^,^^27^ Hyperglycemia is the leading risk factor for DR^13^ as indicated by significantly higher baseline mean HbA1c and glucose measures (Table 1).

### Risk of incidence of diabetes complications in the UKB study

Table 2 shows selected risk factors and their association with the onset of CVD, DKD, and DR in the diabetes (T1D and T2D) cohort. Pooled step-wise variable selection identified different sets of risk factors associated with each complication. Age, male sex, CCI, average SBP, and average glucose were adversely associated with all three DM complications. Cigarette smoking, plasma cholesterol, and uACR are known risk factors for CVD,^28^ and they were adversely associated with the development of CVD in our analysis. Evidence from prior research supports that risk factor variability predicts the development of diabetes complications^29^^,^^30^ in addition to their mean levels. For CVD, consistent with prior research, SBP, HDLc, and uACR variability were associated with an increased risk of CVD.^31^ Higher HDLc was reported protective of CVD onset (HR 0.70, CI: 0.60–0.83).^32^ Both mean and variability of uACR were associated with the development of DKD. Interestingly, for DR, both mean and glucose variability were selected as associated risk factors.^30^^,^^33^^,^^34^ T2D PRS was selected for DR and was estimated to increase the risk of developing DR (HR = 1.04, CI: 1.01–1.08). When we restricted the analysis to T2D subjects, most of the same variables were selected with similar hazard ratios and *P* values (Supplementary Table S6). We note that the estimated effects’ directions of TC and LDL are protective. The possible explanation is participants with DKD and DR tend to have lower TC and LDL levels than those who do not have the condition, potentially due to a higher proportion using cholesterol-lowering medication.

**Table 2.** Risk factors associated with the onset of cardiovascular disease, diabetic kidney disease, and diabetic retinopathy for those with European ancestry within the diabetes cohort

	CVD (1937/9064)^a^		DKD (1945/2650)^a^		DR (3063/2544)^a^	
Risk factor	HR (95% CI)	*P*	HR (95% CI)	*P*	HR (95% CI)	*P*
Age	1.05 (1.04–1.06)	<.001	1.05 (1.04–1.06)	<.001	1.01 (1.01–1.02)	<.001
Sex	2.02 (1.80–2.25)	<.001	0.96 (0.86–1.06)	.418	1.05 (0.98–1.13)	.183
ISCED	0.81 (0.74–0.89)	<.001				
Ever smoked	1.26 (1.15–1.39)	<.001	1.21 (1.09–1.34)	<.001		
MET			0.94 (0.89–1.00)	.038	0.96 (0.92–1.00)	.03
PRS					1.04 (1.01–1.08)	.025
BMI	1.03 (1.02–1.04)	<.001	1.03 (1.02–1.04)	<.001		
CCI	1.26 (1.22–1.30)	<.001	1.14 (1.10–1.18)	<.001	1.04 (1.01–1.07)	.006
CV SBP	1.08 (1.04–1.13)	<.001				
CV DBP			0.92 (0.88–0.97)	.004	0.96 (0.92–0.99)	.015
CV glucose					1.08 (1.03–1.14)	.004
CV HDLc	1.10 (1.05–1.16)	<.001			1.04 (1.00–1.09)	.048
CV eGFR	1.08 (1.02–1.14)	.009				
CV UACR	1.05 (1.00–1.11)	.032	1.21 (1.14–1.29)	<.001		
Mean SBP	1.01 (1.01–1.02)	<.001	1.01 (1.00–1.01)	<.001	1.01 (1.00–1.01)	<.001
Mean DBP	0.97 (0.97–0.98)	<.001			0.99 (0.98–0.99)	<.001
Mean glucose	1.03 (1.01–1.04)	<.001	1.03 (1.01–1.05)	<.001	1.04 (1.03–1.06)	<.001
Mean Chol.	1.10 (1.04–1.15)	<.001	0.83 (0.78–0.88)	<.001		
Mean Trig.			1.08 (1.03–1.14)	.004		
Mean HDLc	0.70 (0.60–0.83)	<.001				
Mean LDLc					0.84 (0.80–0.89)	<.001
Mean eGFR			0.98 (0.97–0.98)	<.001		
Mean UACR	1.05 (1.01–1.08)	.007	1.27 (1.16–1.40)	<.001		

*Note*: We employed the Cox proportional hazards model and a pooled step-wise variable selection procedure to simultaneously analyze imputed data and select important variables that are associated with major diabetes complications outcomes. Our base model included sex, and age, which were not subject to variable selection. Additionally, we included smoking status, BMI, self-reported medication status (insulin, blood pressure, and cholesterol drug), CCI, ISCED level, MET, polygenic risk scores (type 1 and type 2), and summary statistics of biomarker trajectories including average and CV of SBP, DBP, LDLc, HDLc, total cholesterol, glucose, eGFR and urine ACR levels. The variables that were not selected do not appear in this table. The values of MET, average of urine ACR levels, CV of all biomarker levels, and type 1 and type 2 polygenic risk scores were standardized.

CVD: cardiovascular disease; DKD: diabetic kidney disease; DR: diabetic retinopathy; eGFR: estimated glomerular filtration rate; ISCED: International Standard Classification of Education, dichotomous variable, 1 if ISCED level was greater than 2 and 0 otherwise; Smoked, dichotomous variable, 1 if a subject has ever smoked and 0 otherwise; MET: metabolic equivalents to resting state (h/week); BMI: body mass index (kg/m^2^); CCI: Charlson Comorbidity Index; CV, coefficient of variation; Chol: total cholesterol (mmol/l); Trig: triglycerides (mmol/l); HDLc: high-density lipoprotein cholesterol (mmol/l); LDLc: low-density lipoprotein cholesterol (mmol/dl); SBP: systolic blood pressure (mm Hg); DBP: diastolic blood pressure (mm Hg); eGFR: estimated glomerular filtration rate (ml/min/1.73 m^2^); uACR: urine albumin to creatinine ratio (g/mmol); PRS: type 2 diabetes polygenic risk score.

a Number of cases and controls that were included in the model.

**Figure 5.**
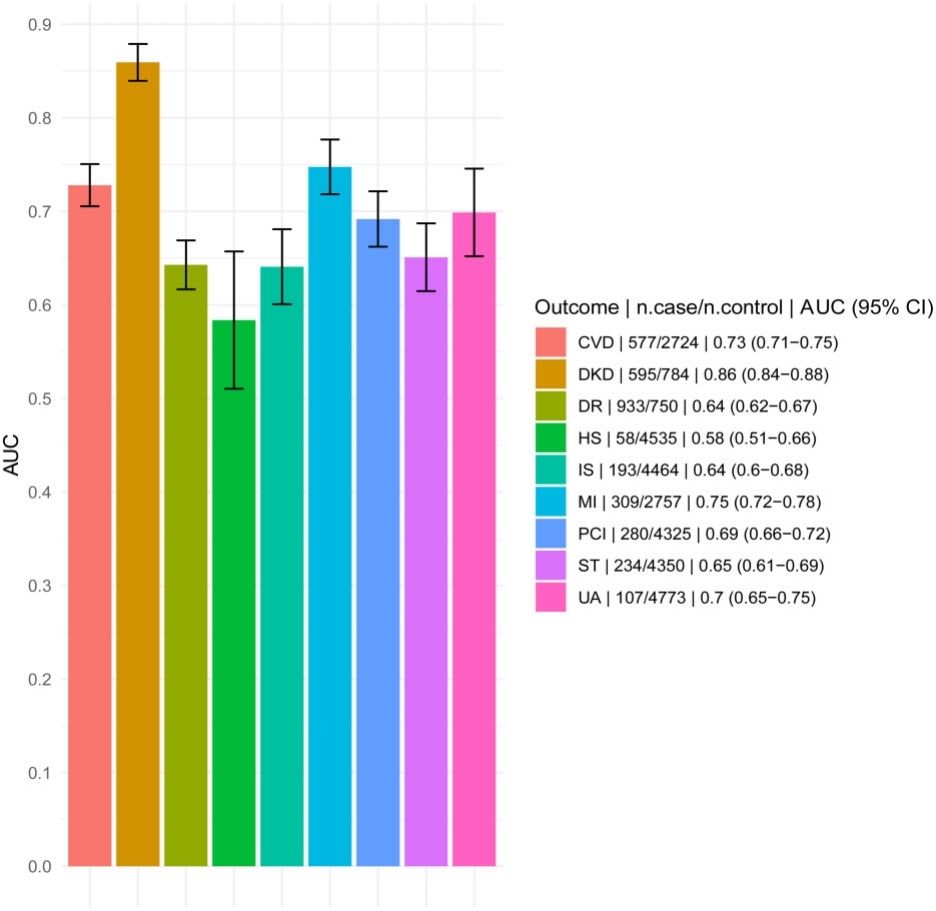
Comparison of prediction performance of risk scores as measured by area under the receiver operating characteristic (ROC) curve (AUC). “n.case/n.control” refers to the number of cases and controls included in the validation data. CVD: cardiovascular disease; DKD: diabetic kidney disease; DR: diabetic retinopathy; HS: hemorrhagic stroke; IS: ischemic stroke; MI: myocardial infarction; PCI: percutaneous coronary intervention; ST: stroke; UA: unstable angina.

**Figure 6.**
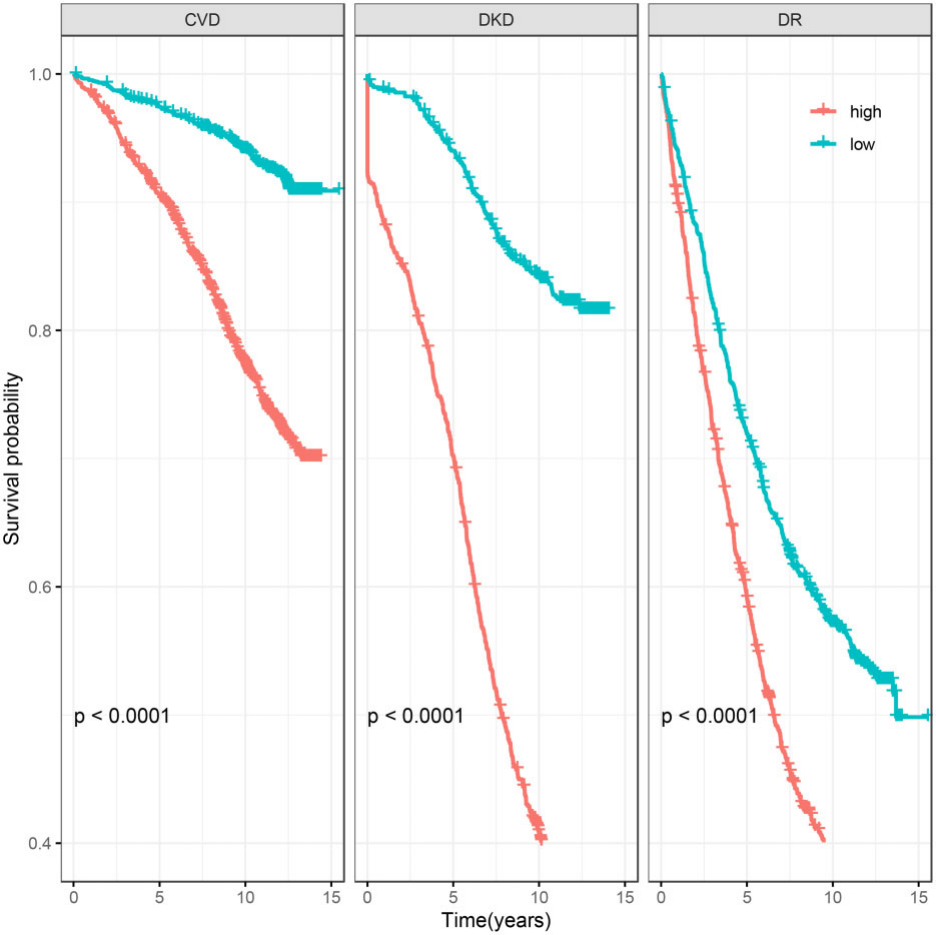
Kaplan–Meier (KM) curves comparing the probability of developing major diabetes complication outcomes between low- and high-risk groups among European validation cohort of diabetes subjects. Individuals were assigned to high-risk group if their risk scores were greater than the median risk score and to low-risk group if otherwise. CVD: cardiovascular disease; DKD: diabetic kidney disease; DR: diabetic retinopathy.

The risk prediction performance of each outcome was assessed using validation data. Prediction performance was measured by AUC (Figure 5). Among the major complication outcomes, DKD showed the highest AUC (0.86, 95% CI: 0.84–0.88), followed by CVD (0.73, 95% CI: 0.71–0.75) and DR (0.64, 95% CI: 0.62–0.67). These results are consistent with the prediction AUC of standard risk prediction models using cohort studies, suggesting some equivalence in EHR.^35^Supplementary Table S7 shows the selected variables using the pooled step variable selection procedure with a training dataset for each outcome. The AUCs for these models applied to non-European participants were similar (Supplementary Figure S3). The KM curves comparing the probability of developing major diabetes complications between low- and high-risk groups (with respect to a median risk score) are shown in Figure 6. For each outcome, the high-risk group showed a significantly higher probability of progressing to develop DM complications than the low-risk group. The KM curves of the other outcomes can be found in Supplementary Figure S1.

## DISCUSSION

In this paper, we provide a framework to showcase phenotyping for the study of disease progression using UKB data. We focus on the definition of events, curation of event dates, and their censoring time. We provide a pipeline to phenotype cardiovascular and microvascular complications after the diagnosis of diabetes. We use diabetes complications as an example because their phenotyping is heterogeneous, as diabetes disease progression is due to hyperglycemia exposures and many other risk exposures.^13^ Beyond phenotyping diabetes complications, we also curated traditional risk factors longitudinally. Our pipeline enables streamlined calculation of the incidence event rate, time-varying risk factors underlying disease progression, and time-to-event analyses. Using curated phenotypes, we assessed the effects of known risk factors for DM complications. Existing algorithmic definitions provide rules and procedures to phenotype a disease of interest. However, a consistent and organized framework to produce all necessary data ingredients is lacking. This step is the bottleneck in applying certain algorithmic definitions for phenotyping. Our platform comprehensively produces data ingredients in a consistent and modular way to allow the efficient application of specific phenotyping algorithms. Using diabetes and its complications as an example, we show how the platform can generate needed data ingredients from UKB-provided resources, including predefined fields associated with diseases and linked primary care data. In contrast, Eastwood et al applied sequential, multi-level rules to self-report and nurse interview data to identify subjects with indications of diabetes.^36^

Our analysis results are consistent with Pittsburgh Epidemiology of Diabetes Complications (EDC) Study and EDIC study where a similar definition of CVD phenotype was employed.^37^ They reported that accounting for other risk factors, higher DBP is protective against developing CVD, although with a modest hazard ratio (ie, HR = 0.97, CI: 0.97–0.98, *P* value <.001). Similar observations were reported before.^31^ In our analysis, uACR strongly predicts future DKD. Indeed, albuminuria is the most prominent symptom of essentially all kidney diseases.^38^ Interestingly, we reported that uACR is an independent risk factor for the onset of CVD. It indicates that any degree of albuminuria is a risk factor for CVD events in individuals with DM; the risk increases with the uACR, starting well below the microalbuminuria cutoff. Screening for albuminuria can identify people at high risk for CVD events.^39^

We applied strict filtering criteria to filter patients in the risk set. This means computed incidence rates should be interpreted in the context of subjects with primary care data available (DR and DKD) and without prior conditions. These exclusion criteria may be removed according to studies’ needs. CVD outcomes were mostly curated from hospital records; to keep a larger sample size, we did not require all the participants to have primary care data. Thus, fewer repeated measures of biomarkers were involved in capturing their variability. Although we focused on macrovascular and microvascular events as diabetes complications, the definitions can be used broadly. We primarily considered right censoring. However, when definitive disease ascertainment is unavailable, both time-to-event outcomes and covariates are subject to complex censoring mechanisms. In the Supplementary Materials, we discussed other types of censoring mechanisms (left censoring and interval censoring).

Our approach has a few limitations. We used an indirect method to evaluate event definitions rather than an independent “gold” standard, for example, chart review. The phenotypes created and evaluated in this manuscript are predominantly diabetes and CVDs related. Experienced clinicians in our team provided valuable input for each phenotyping pipeline to maximize the information extracted from EHRs and minimize the risk of mischaracterizing patients’ disease onset and progression. Disease or syndrome’s phenotypes are often represented by hundreds of terms. As such, while the method described in our manuscript yields robust results for the phenotype use cases presented here, additional conditions or terms may still be necessary to refine the phenotypes. Users should incorporate them when seeing fit. Further research is required to ascertain other disease statuses. Finally, an important contributor to variability in risk factors is the effect of changes in dosing or types of medications, which are often adapted to biomarker levels. As DM management typically requires many medications, it is particularly relevant to capture changes in medications over time accurately. We defer appropriately capturing and incorporating medication use into future research.

## CONCLUSION

We provide a unique resource to showcase time-to-event outcome phenotyping for the study of disease progression using UKB data. Our phenotyping framework, detailed terms curated, and analysis code are all publicly available to facilitate reproducibility and transparency.

## Supplementary Material

ooad006_Supplementary_Data

## Data Availability

The source codes of the pipeline producing the analysis results are available online at https://github.com/dohyunkim116/ukbiobank-phenotyping-book/tree/analysis-pipeline. The source codes to reproduce the phenotyping book is available online at https://github.com/dohyunkim116/ukbiobank-phenotyping-book/tree/main. The UK Biobank data are available to investigators through an application for access (https://www.ukbiobank.ac.uk/enable-your-research/apply-for-access). Data from UK Biobank are approved through application ID: 48152.
