# Supplementary material for: A platform for phenotyping disease progression and associated longitudinal risk factors in large-scale EHRs, with application to incident diabetes complications in the UK Biobank
Source: JAMIA Open. 2023 Feb 9;6(1):ooad006. doi: 10.1093/jamiaopen/ooad006 (PMC9912368; doi:10.1093/jamiaopen/ooad006)
Supplement: ooad006_Supplementary_Data [file ooad006_supplementary_data.docx]

**Supplementary Materials**

Table of Contents

[Supplementary Text 2](#_Toc115878878)

[Table S1. Summary of UKB fields used to phenotype diabetes and complication outcomes 5](#_Toc115878879)

[Table S2. Fields and terms associated with outcome definition. 6](#_Toc115878880)

[Table S3. Fields and terms associated with biomarkers or covariates 11](#_Toc115878881)

[Table S4. Fields used to determine censoring dates 12](#_Toc115878882)

[Table S5. Self-reported ethnicity categorization 13](#_Toc115878883)

[Table S6. Risk factors associated with the onset of cardiovascular disease, diabetic kidney disease, and diabetic retinopathy for Type 2 diabetes cohort of European ancestry 14](#_Toc115878884)

[Table S7. Selected variables using pooled step variable selection procedure with training data of Europeans with diabetes for each outcome 15](#_Toc115878885)

[Table S8. Selected variables using pooled step variable selection procedure with training data of Europeans with Type 2 diabetes for each outcome 16](#_Toc115878886)

[Figure S1. Kaplan-Meier curves comparing survival probability of experiencing major diabetes complication outcomes between low- and high-risk groups 18](#_Toc115878887)

[Figure S2. Comparison of prediction performance of risk scores as measured by AUC for Type 2 diabetes subjects 19](#_Toc115878888)

[Figure S3. Comparison of prediction performance of risk scores as measured by AUC for non-European diabetes subjects 20](#_Toc115878889)

# **Supplementary Text**

1. **Diabetes Mellitus (DM)**

We use the primary care data and the UKB assessment center data to categorize diabetes into Type 1 or Type 2 diabetes. We categorized a type of diabetes into one of three types which included Type 1, Type 2, and Uncertain. For UKB assessment center data, this information was provided in fields *1300706, 130708,* and *130714* (Type 1, Type 2, and unspecified, respectively). For the primary care data, we first identify the codes that indicate a certain type of diabetes by searching pre-defined keywords in the diabetes-specific dictionary. The pre-defined keywords that map to either Type 1 or Type 2 diabetes are summarized below:

| **Type** | **Keywords** |
| --- | --- |
| Type 1 | type1, type1[$-], juvenile, insulin-dependant, insulin dependent |
| Type 2 | type2, type II, adult onset, non[-]insulin[-]dep, NIDD, maturity onset diabetes mellitus |

Next, using these codes, we search the primary care data of subjects with clear indications of diabetes to mark which events are associated with a certain type of diabetes. Finally, we resolve a type of diabetes for each subject identified from the UKB assessment center data and the primary care data by the following rules:

Assume we only work with unique types of categories. For example, if we have two events from the UKB assessment center data and the primary care data that are both associated with Type 1 diabetes, then we assume there is only one unique event associated with Type 1 diabetes.

1. If there is only one unique type across all events, then we take that type as the type of diabetes for a subject, which is either Type 1, Type 2 or Uncertain.
2. If there are exactly two unique types, then we take the type to be either Type 1 or Type 2 unless the two unique types are Type 1 and Type 2 which indicate Uncertain type.
3. If there are more than two unique types, then the subject has Uncertain type of diabetes.
4. **Myocardial Infarction**

Myocardial infarction (MI) was identified by the ICD-10 code family I21 (Acute MI). The main source for this code was UKB’s field 131298 (Date I21 first reported). This field gathers information from hospital admissions, death records, primary care, and self-reported outcomes from surveys taken at UKB assessment centers at initiation into the study and maps them to three-digit ICD-10 categories. In order to obtain the most up-to-date information, we also gather this ICD-10 code family directly from hospital admission and death records. We also included events of MI identified through UKB’s algorithmically defined outcome (field 42000), previously described (resource 461). Patients in the risk set were required to have no evidence of certain cardiovascular diseases (**Table S2**).

1. **Percutaneous Coronary Intervention (PCI)**

PCI was identified through OPCS4 codes K40, K41, K42, K43, K44, K45, K46, K483, K49, K501, K75, K76, and UKB self-report codes 1070 (Coronary Angioplasty) and 1095 (Coronary bypass grafts). Patients in the risk set were required not to have self-reported any non-coronary revascularization procedures (**Table S2**).

1. **Unstable Angina**

Unstable Angina was identified by the ICD-10 code I200, extracted from hospital admissions and death records.

1. **Ischemic Stroke**

Ischemic strokes were identified in a manner like MI, using a combination of UKB’s first occurrence field 131366 (Date I63 first reported (cerebral infarction)), the algorithmically defined outcome for ischemic stroke (field 42008), and the ICD-10 code I63 in hospital admission or death records. Patients in the risk set were required to have no evidence of cerebrovascular disease (ICD10 codes I6*, G45*).

1. **Hemorrhagic Stroke**

Hemorrhagic strokes were identified using a combination of UKB’s first occurrence fields 131360 (Subarachnoid Hemorrhage), 131362 (Intracerebral Hemorrhage), 131364 (Other non-traumatic Hemorrhage), as well as algorithmically defined outcomes in fields 42010 (Intracerebral Hemorrhage), 42012 (Subarachnoid Hemorrhage), and the ICD-10 code prefixes I60, I61, or I62 in hospital admission or death records. Patients in the risk set were required to have no evidence of cerebrovascular disease (ICD10 codes I6*, G45*).

1. **Stroke**

Stroke was taken to be the first occurrence of either ischemic or hemorrhagic stroke or of unspecified stroke or other types of strokes (specified but not ischemic or hemorrhagic) via UKB fields 42006 (algorithmically defined stroke), 131368 (unspecified stroke), ICD10 code I64, or self-reported condition code 1081. Patients in the risk set were required to have no evidence of cerebrovascular disease (ICD10 codes I6*, G45*).

1. **CVD death**

CVD death was defined as a primary cause of death falling under ICD10 codes I* (diseases of the circulatory system).

1. **Composite CVD**

A composite event was analyzed, which consisted of either MI, PCI, Unstable Angina, Ischemic Stroke, or CVD death. The first date of CVD was taken as the first date of any of these events. Patients in the risk set were required to satisfy all of the conditions for each component outcome.

1. **Diabetic Eye Disease**

Diabetic eye disease was determined using the ICD10 codes E1*.3 (Diabetes Mellitus with Ophthalmic Complications), H36.0 (Diabetic Retinopathy), and H28.0 (Diabetic Cataract), as well as a set of primary care codes listed in **Table S2**. Since most events were identified through primary care data, patients in the risk set were required to have primary care data to reduce misclassification. Patients in the risk set were required not to have any glaucoma, cataract, or non-diabetic/unspecified retinopathy.

1. **Macroalbuminuria/Microalbuminuria**

Urine Albumin:Creatinine Ratio (ACR) was calculated using UKB fields 30700 (urine creatinine), 30500 (urine microalbumin), and 30505 (reason for missing urine microalbumin). ACR above 33.9 was considered macroalbuminuria, while above 3.4 was considered microalbuminuria. In cases where urine microalbumin was below detectable levels, albuminuria status was inferred from urine creatinine where possible.

1. **Diabetic Kidney Disease (DKD)**

DKD was identified through UKB’s algorithmically defined End-Stage Renal Disease (ESRD) (field 42026, previously described), ICD10 codes E1*.2 (Diabetes Mellitus with renal complications), E18[0345] (CKD Stage 3-5, end-stage), N08.3 (Glomerular disorders in diabetes mellitus) in hospital or death records, self-reported diabetic kidney disease, two or more eGFR (EPI creatinine) < 60 mL/min/1.73m^2^, 90 days apart, without any eGFR > 60 in between (from either UK Biobank Assessment Center or primary care data). The date of the first DKD was taken as the first occurrence of any of the codes/events. Patients in the risk set were also required to have primary care data linkage available and not to have micro/macroalbuminuria or a list of exclusion codes. In addition, patients in the risk set were required to have at least 5 years of follow-up since their diagnosis with diabetes, and patients having DKD events were required to have at least 5 years from the diabetes diagnosis and first DKD event in order to preferentially capture diabetes-related kidney disease as opposed to DKD arising from a different etiology.

1. **Interval Censoring**

While our study used participants’ first visit to a UKB assessment center as the index date for survival traits, another possible study design uses the participants’ first diagnosis of DM as the index date. For this design, survival traits may be considered interval-censored if the date of the first diagnosis with DM is unknown. For example, if a DM-related code is recorded at a patient’s first primary care or hospital encounter, their disease status is assumed to precede that encounter, but the duration of disease prior to that encounter is unknown. We assigned right- and interval-censored flags for each patient with DM according to the following scheme:

For each individual in this cohort, we consider the DM diagnosis date to be known if either

- 1. provided by the individual in UKB assessment questionnaire (field 2976) or
  2. inferred from the first occurrence of DM ICD9/10 code in hospital admission record or primary care data AND there are at least 2 prior years of primary care record for that individual without evidence of DM.

We consider the DM diagnosis date to be unknown if

- 1. the answer to the UKB assessment questionnaire is unavailable, AND there is insufficient period of record (less than 2 years before first evidence of DM) in primary care data, OR
  2. scenarios where certain DM complications (Diabetic Retinopathy [DR] or Diabetic Kidney Disease [DKD]) are recorded on the same date as, or are themselves, the first evidence of DM.

If the DM first diagnosis date is unknown and CVD complication has not occurred yet (T_CVD_=∞), then the time-to-event is lower bounded by (L_i_ = max(last UKB assessment time of this individual, last admission record date, last primary care record date) - T_DM_). The outcome for such patients is right-censored (Ri = ∞).

If the DM first diagnosis date is unknown (DM diagnosis date before T_DM_) and CVD complication has already occurred, then the time-to-event is lower bounded by (L_i_ = T_CVD_ - min(first primary care or admission record time, T_DM_)) and upper bounded by (R_i_ = T_CVD_ - birth date). The outcome for such patients is interval-censored.

If the DM first diagnosis date is known and CVD complication has not occurred yet (T_CVD_= ∞), then the time-to-event is lower bounded by (L_i_ = max(last UKB assessment time of this individual, last admission record date, last primary care record date) - T_DM_). The outcome for such patients is right-censored (R_i_ = ∞).

If the DM first diagnosis date is known (DM diagnosis date = T_DM_) and CVD complication has already occurred, then the time-to-event is exactly observed (L_i_ = R_i_ = T_CVD_ - DM diagnosis date).

# **Table S1. Summary of UKB fields used to phenotype diabetes and complication outcomes**

| **Field type** | **UKB Fields** |
| --- | --- |
| First occurrence | All of the fields in *Category 1712* |
| Algorithmically defined | *42000, 42008, 42010, 42012, 42006, 42026* |
| ICD10 | *41270, 41280, 40001, 40002, 40000* |
| OPCS4 | *41272, 41282* |
| Self-reported illness | *20002, 20008* |
| Self-reported operation | *20004, 20010* |
| Primary care codes | *42040* |
| Custom-defined^1^ | *5901.0.0, 5901.1.0, 5901.2.0, 5901.3.0* |

^1^: This is a custom-defined field for capturing self-reported diabetic retinopathy events.

# **Table S2. Fields and terms associated with outcome definition.**

| Conditions | Type | UKB fields | Terminology | Terms | Data Source(s) |
| --- | --- | --- | --- | --- | --- |
| Diabetes Mellitus | Case status | 130706, 130708, 130714 | Read v2/CTV3 | 1434., 14P3., 2BBF., 2BBk., 2BBl., 2BBL., 2BBo., 2BBP., 2BBQ., 2BBR., 2BBS., 2BBT., 2BBV., 2BBW., 2BBX., 2G5d., 2G5e., 2G5F., 2G5G., 2G5H., 2G5J., 2G5K., 2G5L., 2G5V., 2G5W., 661M4, 661N4, 66A1., 66A3., 66A4., 66A5., 66Aa., 66AG., 66AH[.0], 66Ai., 66AI., 66AJ[.1z], 66AK., 66AL., 66AN., 66Ao., 66AR., 66At[01], 66Au., 66Av., 66AV., 66o6., 7276., 7L198, 8B3l., 8BL2., 8CE02, 8CR2., 8CS0., 8HBG., 8HBH., 9b920, C10.., C100[.01z], C101[.01yz], C102., C103[.0z], C104[.1z], C105[.1z], C106[.01yz], C107., C108[.0135789], C109[.01234679CEJK], C10C., C10D., C10E[.78DFHKLMPQ], C10F[.0679BCEHJKLMNQRS], C10yz, C10z., C110., Cyu2., F1711, F3450, F35z0, F372[.012], F3813, F3y0., F420[.01234678z], F4407, F4640, G73y0, K08yA, K27y7, L180X, M0372, M21yC, M271[012], N030[01], R0542, U6023, X00A[ghjl], X00d[FGI], X30K[klm], X40J[456bIJqru], X5086, X50E7, X50GP, Xa0lK, Xa1J5, Xa4g7, Xaa8r, XaBul, XaBZQ, XaCJ2, XacsY, XaD2T, XaDZH, XaE5[acTUVX], XaEgI, XaEL[PQ], XaEn[opq], XaEV[PST], XaF0[45], XaFm[8AL], XaFn[89], XaFW[GI], XaIe[IJKMRS], XaIf[GIM], XaIP[5k], XaIr[fM], XaIW8, XaIyz, XaIz[0MNQR], XaJ5j, XaJK3, XaJL[ab], XaJlI, XaJO[ghijklno], XaJQp, XaJSr, XaJYg, XaKcS, XaKD[GH], XaKH[hi], XaKSn, XaKwQ, XaKy[WX], XaLSk, XaMhK, XaPmX, XaX3[pq], XaX6[JK], XaXbW, XaXfs, XaXgv, XaXhE, XaXZR, XaYbB, XaYQZ, XaYZP, XC0ca, XE10[EFGHIJ], XE12[8AGIK], XE15k, XE18r, XE1T[23], XE1V0, XM0q4, XM1Xk, XSETH | Primary care (gp_clinical) |
|  | Date of onset only | 130706, 130708, 130714 | Read v2/CTV3 | 13AB., 13AC., 13B1., 14F4., 1M8.., 2G510, 2G5C., 2G5E., 2G5I., 44Uz., 44V3., 66A.., 66A2., 66A6., 66A7[.01], 66A8., 66A9., 66AA., 66AB., 66Ac., 66AC., 66Ad., 66AD., 66Ag., 66Ah., 66AJ[023], 66Ak., 66Al., 66Am., 66AM., 66An., 66AO., 66Ap., 66AP., 66Aq., 66AQ., 66Ar., 66AS[.0], 66At., 66AT., 66AU., 66Aw., 66AW., 66Ax., 66AY., 66AZ., 671F[01], 68A7., 7L100, 8A12., 8A13., 8BAi., 8BAj., 8BAm., 8CA41, 8CE01, 8CP2., 8H2J., 8H3O., 8H7C., 8H7r., 8Hg4., 9360., 93C4., 9kL.., 9m0.., 9m00., 9N0m., 9N0n., 9N0o., 9N1i., 9N1o., 9N1Q., 9N1v., 9NJy., 9NM0., 9OL.., 9OL1., 9OL3., 9OLA., 9OLE., 9OLF., 9OLZ., C102z, C1074, C108z, C109[5F], C10ER, C10F[8F], C10J., C10y., C110[01z], C1111, C112[.01z], C116., C1A.., C3070, Cyu20, Cyu30, f1..., f2..., f25.., f29.., f2A.., f2Ay., F3745, fw2.., Kyu03, L1806, R1057, R10D., Ryu8A, TJ23[034AB], Ua1M[de], x006f, x008T, X00Am, x01L[bcf], X305t, X40J[23ceEntxz], X40K[1F], X50GO, X74WS, X7739, X795r, Xa0k8, Xa2h[79A], Xa3ee, Xa5XZ, Xa6Tk, Xa9Ao, XaaC[RV], Xaag1, XaAk4, XaaNP, Xabhn, XaBP1, XabQM, XaBU[89], XaBu[hin], XaBw[jk], XaBY7, Xac0x, XaC4i, XaCES, XacIu, XaE4q, XaEHx, XaEJQ, XaFmM, XaFn7, XaFv[jk], XaFxf, XaIe[HLT], XaII[ej], XaIQS, XaIrL, XaIy[tu], XaJEN, XaJOO, XaJUH, XaKa[LM], XaKb[Hu], XaKHD, XaKj[XYZ], XaKvW, XaKzP, XaL2E, XaLMy, XaMdq, XaMhJ, XaN1z, XaNHW, XaOPu, XaOzL, XaP5E, XaP63, XaPa[acd], XaPbv, XaPem, XaPQH, XaQdT, XaQGH, XaR7E, XaRGt, XaX3o, XaX9n, XaXfW, XaXH6, XaXOw, XaXZv, XaYQ[dfhiX], XaYwh, XaZig, XaZLX, XaZS6, XE12M, XE1Uy, XE28s, XE2N[de], XM1Rs, XS7Nb, Y0022, Y09df, Y09e[0123], Y0c4[89], Y0c50, Y0c8b, Y0c9b, Y0cbb, Y0cc[23], Y0fe[34], Y102[679], Y1109, Y1123, Y1206, Y128[68], Y1290, Y1585, Y1604, Y2622, Y3044, Y3579, Y361[236], Y6850, Y9993, YX128, ZV6D[AB] | Primary care (gp_clinical) |
| Myocardial infarction (MI) | Case status and date of onset | 131298, 131300, 42000 | ICD-10 | I2[123] | Hospital admissions  Death record |
|  | Control exclusion |  | ICD-10 | I[23567]* | Hospital admission  Death records |
|  |  |  | UKB Data coding 5 | 107[146789], 1080, 110[25789], 1110, 1471, 1489, 149[02], 158[456789], 159[012] | Self-reported operations (Field 20004) |
|  |  |  | UKB Data coding 6 | 1082, 1086, 1069, 109[6789], 110[014], 1523, 155[34] | Self-reported illness (Field 20002) |
| Unstable angina | Case status and date of onset |  | ICD-10 | I20.0 | Hospital admissions  Death record |
| Ischemic stroke | Case status and date of onset | 131366, 42008, 2002 | ICD-10 | I63 | Hospital admissions  Death record |
|  |  |  | UKB Data coding 6 | 1583 | Self-reported illness (Field 20002) |
|  | Control exclusion |  | ICD-10 | G4[56], I[23567] | Hospital admissions  Death record |
|  |  |  | UKB Data coding 5 | 107[146789], 1080, 110[25789], 1110, 1471, 1489, 149[02], 158[456789], 159[012] | Self-reported operations (Field 20004) |
|  |  |  | UKB Data coding 6 | 1082, 1086, 1069, 109[6789], 110[014], 1523, 155[34] | Self-reported illness (Field 20002) |
| Hemorrhagic stroke | Case status and date of onset | 131360, 131362, 131364, 42010, 42012, 20002 | ICD-10 | I6[012] | Hospital admissions  Death record |
|  |  |  | UKB Data coding 6 | 1086 | Self-reported illness (Field 20002) |
|  | Control exclusion |  | ICD-10 | G4[56], I6[56789] | Hospital admissions  Death record |
|  |  |  | UKB Data coding 6 | 1082 | Self-reported illness (Field 20002) |
| Any Stroke | Case status and date of onset | 42006, 131368, 20002 | ICD-10 | I6[01234] | Hospital admissions  Death record |
|  |  |  | UKB Data coding 6 | 1086, 1583, 1081 | Self-reported illness (Field 20002) |
|  | Control exclusion |  | ICD-10 | G4[56], I[23567] | Hospital admissions  Death record |
|  |  |  | UKB Data coding 5 | 107[146789], 1080, 110[25789], 1110, 1471, 1489, 149[02], 158[456789], 159[012] | Self-reported operations (Field 20004) |
|  |  |  | UKB Data coding 6 | 1082, 1086, 1069, 109[6789], 110[014], 1523, 155[34] | Self-reported illness (Field 20002) |
| Percutaneous Coronary Intervention (PCI) | Case status and date of onset | 20004 | OPCS4 | K4[01234569], K483, K501, K7[56] | Hospital admissions |
|  |  |  | UKB Data coding 5 | 1070, 1095 | Self-reported operations (Field 20004) |
|  | Control exclusion |  | ICD-10 | I[23567] | Hospital admissions  Death record |
|  |  |  | UKB Data coding 5 | 107[146789], 1080, 110[25789], 1110, 1471, 1489, 149[02], 158[456789], 159[012] | Self-reported operations (Field 20004) |
|  |  |  | UKB Data coding 6 | 1082, 1086, 1069, 109[6789], 110[014], 1523, 155[34] | Self-reported illness (Field 20002) |
| Diabetic Eye Disease | Case status and date of onset | 5901 | ICD-10 | E1[0134]3, H36.0, H28.0 | Hospital admissions  Death record |
|  |  |  | Read v2/CTV3 | 2BBF., 2BBk., 2BBl., 2BBL., 2BBo., 2BBP., 2BBQ., 2BBR., 2BBS., 2BBT., 2BBV., 2BBW., 2BBX., 7276., 8HBG., 8HBH., C105[.1z], C108[17], C109[16E], C10E[7FP], C10F[6EQ], F420[.01234678z], F4407, F4640, X00d[FGI], XaBul, XaD2T, XaE5[cTUVX], XaEV[PST], XaFm[8A], XaIP[5k], XaIW8, XaJL[ab], XaJlI, XaJO[ghijklno], XaJQp, XaJSr, XaKcS, XaKD[GH], XaXfs, XE12G, XE18r, Y8616, Y8657 | Primary care (gp_clinical) |
|  |  |  | UKB Data coding 6 | 1276 | Self-reported illness (Field 20002) |
|  | Control exclusion | 20002, 20004 | ICD-10 | H2[568], H3[456], H4[02] | Hospital admissions  Death record |
|  |  |  | Read v2/CTV3 | 1481., 1482., 1483., 14N9., 14NA., 14NC., 1JF.., 22E5., 2BB2., 2BB3., 2BB4., 2BB5., 2BB6., 2BB7., 2BBa., 2BBG., 2BBH., 2BBm., 2BBn., 2BBN., 2BBs., 2BBY., 2BT0., 2BT1., 2F2.., 2F22., 2F2Z., 5831., 5B4.., 5B42., 668.., 66B4., 66T1., 72513, 72522, 7259[012], 7263[.0], 7264., 7266[.1], 7267[36], 727.., 7271[.1yz], 7272[.012456789], 7273[.045yz], 7275., 7277[.026], 727B1, 727y., 727z., 8HBD., 8LC0., B7J03, F101[25], F4042, F41.., F410[.03567z], F411[.0134z], F412., F413[.0124z], F41y[.0z], F41z., F42.., F421[.013456789ADEFHz], F422[.01z], F423[.012356789Az], F424[.1], F425[.012346789Az], F426[.01236z], F427[.3489ABCGHJKLz], F42y[.01345679BCDz], F42z., F43.., F430[.045z], F431., F432[.1z], F433[.02z], F4421, F45.., F450[.012z], F451[.01245z], F452[.0123z], F454[.01], F455[01], F456[.26z], F45y[.02z], F45z., F46.., F460[.01234567z], F461[.02345789yz], F463[.24z], F464[.46z], F465[.03], F466., F46y[.z], F46z[.0], F4A24, F4B55, F4G2., F4H14, F4K2D, FyuBD, FyuE[01], FyuF[.0345789], FyuG[.0], k8..., P32.., P33.., P330., P331[.01z], P332[.1], P333., P33y0, P33z., P351., P352., P354., P355[.0z], P3570, PK61., R1411, X00c[9ltUvxz], X00d[01234bBCdeEfgkKmnoOpqrTwxY], X00e[cefFimNrs], X00X[eFgGhHIjprtuZ], X75k[kmX], X75l[abcgjnorUWZ], X75m[389AbBDgKLmMNopPTWy], X75n[0138BCDeKNTW], X76Fh, X77s[XYZ], Xa05T, Xa3f[DE], Xa3m[pqu], Xa3q2, Xa9B[IJKLMNPQT], XaBL[56OP], XaBum, XaBY[NO], XaCKF, XaCMW, XaD2a, XaE0[ablYZ], XaE1x, XaE5[bdIjJlNoqQrR], XaE6T, XaE7[TUVY], XaEUK, XaEV[BCpW], XaF1D, XaF7p, XaF9[DI], XaFa[pv], XaFTm, XaG29, XaIsW, XaJT[LMN], XaKD[IJ], XaL4[259AQ], XaL5[8ACLmMnoOPRX], XaLWk, XaMsj, XaNcY, XaNko, XaX5S, XaYiR, XC0a1, XC0sa, XE0B[IRSTU], XE0K[qs], XE15[suvwxy], XE16[023], XE18[fhjp], XE1im, XE1Ju, XE1T0, XE2a8, XE2RP, XM04[opqs], XM0nB, XM1I0, XS29G, Y0c4[4567], Y122[0145], Y123[67], Y124[67], Y219[346], Y2539, Y586[67], Y6685, Y7929, Y8627, Y893[23678], ZV456 | Primary care (gp_clinical) |
|  |  |  | UKB Data coding 6 | 127[578], 128[12], 1527, 153[08] | Self-reported illness (Field 20002) |
|  |  |  | UKB Data coding 5 | 143[4567] | Self-reported operations (Field 20004) |
| Chronic/Diabetic Kidney Disease | Case status and date of onset | 30700, 30500, 30505 | ICD-10 | E1[0134].2, N08.3, N18.[0345] | Hospital admissions  Death record |
|  |  |  | Read v2/CTV3 | 1Z12., 1Z13., 1Z14., 1Z15., 1Z16., 1Z1a., 1Z1b., 1Z1B., 1Z1C., 1Z1D., 1Z1E., 1Z1F., 1Z1G., 1Z1H., 1Z1J., 1Z1K., 1Z1L., 1Z1T., 1Z1X., 1Z1Z., C104[.1z], C1080, C109[0C], C10E[DKL], C10F[0CLM], D2150, K01.., K01z., K05.., K050., K053., K054., K055., K08yA, K0D.., K190X, Kyu03, Kyu21, Kyu5G, X30J[02], X30K[klm], XacA[29bdeMNOVWX], XaCLy, XaF0[45], XaIyz, XaIz[0MNQR], XaLH[IJK], XaNb[no], XaO3[tuvwxyz], XaO4[012], XC0ca, XE0df, XE10G, XM19A, Y1585 | Primary care (gp_clinical) |
|  |  |  | UKB Data coding 6 | 1607 | Self-reported illness (Field 20002) |
|  | Control exclusion | 20002, 20004 | ICD-10 | N[012], Z49, Z99.2 | Hospital admissions  Death record |
|  |  |  | Read v2/CTV3 | 14S2., 1Z1.., 1Z10., 1Z11., 1Z17., 1Z18., 1Z19., 1Z1A., 1Z1M., 4678., 661M2, 661N2, 66i.., 6AA.., 7A60[126z], 7A61[14], 7B00[.12z], 7B015, 7L1A[.012], 7L1B[.01z], 7L1C0, C341., C373[46], D215., G22.., G222., G7521, G760., J624., K0..., K010., K011., K012., K013., K014., K015., K016., K017., K019., K01A., K01w0, K01x[04], K02.., K020., K021., K022., K02y[.02z], K03.., K032[06y], K03U., K03X., K03y[.z], K03z., K051., K052., K06.., K072., K08y0, K0A.., K0A17, K0A2[358], K0A3[.17], K0A4[13], K0A5[.12], K0y.., K0z.., K11.., K111., K112., K113., K11X., K11z., K136., K13yB, Kyu0[08], Kyu1F, Kyu2., PD13., PD23., R110[.013z], SP056, SP083, TB001, X018[MNT], X01A[LP], X30H[gu], X30I[24dFGLMnoQRW], X30Kz, X30L[2r], X30M[5cnNP], X30Q[12], X40bz, X40c3, Xa0Ex, Xa0HK, Xa1dw, Xa1uD, Xa24a, Xa33[cg], Xa8S7, Xa9zl, Xaa5[fT], Xaa6o, Xaa7I, XaC2Z, Xac9y, XacA4, XaCLx, XaCMe, XaE6[pq], XaE9T, XaLH[GH], XaLiH, XaMGE, XaMJD, XaMKM, XaMMt, XaO3[pqrs], XaOmL, XaXgX, XaXhq, XaXTz, XaXYe, XaYb9, XaYZW, XC09o, XC0sw, XE0d[abcdgZ], XE0F[hjv], XE0f5, XE0J[fg], XE2u6, XM08q, XM19[8gLtuZ], XM1AT, XM1C[rt], Y1365, Y5355, Y7746, Y8576, Y8669, Y8670, ZV420, ZV451, ZV561, ZVu3G | Primary care (gp_clinical) |
|  |  |  | UKB Data coding 6 | 119[234], 1519, 1520, 160[89] | Self-reported illness (Field 20002) |
|  |  |  | UKB Data coding 5 | 1195, 1487 | Self-reported operations (Field 20004) |
|  |  |  | OPCS4 | M0[123], X4[012] | Hospital admissions |

# **Table S3. Fields and terms associated with biomarkers or covariates**

| **Biomarker/Covariate** | **UKB fields** | **Primary care terms** |
| --- | --- | --- |
| Platelets | 30080 |  |
| Urine albumin | 30500 | 46N4, XE2eI, 46N8., 46W[.01], XE2bw |
| Urine creatinine | 30510 | 46M7 |
| Urine albumin to creatinine ratio |  | 46TC, XE2n3, X773Y, 46TD, XE2n4 |
| Blood albumin | 30600 |  |
| Blood creatinine | 30700 | 44J3[.0123z], 44J[CDF], XE2q5, XaERc, XaERX, XaETQ, 4Q40., X771Q |
| Cholesterol | 30690 | 44P[.12349HJKZ], XE2eD, XSK14, XaFs9, XaIRd, XaJe9, XaLux |
| Cystatin C | 30720 |  |
| Blood glucose | 30740 | 44[fg][0.], 44TA, XM0ly |
| Fasting glucose |  | 44[fg]1 |
| HbA1c | 30750 | XaPbt, XaERp, X772q, 42W[12345Z.]., 44TB. |
| High-density lipoprotein | 30760 | 44d[23], X772M, 44P[5BC], XaEVr |
| Low-density lipoprotein | 30780 | 44d[45], 44P[6DE], XaEVs |
| Triglycerides | 30870 | 44e, 44Q, X772O, XE2q9 |
| Systolic blood pressure | 4080, 93 | 246[.cdgABCDEFGJNPQRSTVWXY012345679], XaF4[abFLOS], XaJ2[EFGH], XaKF[xw], G20 |
| Diastolic blood pressure | 4079, 94 |  |
| BMI^1^ | 21001, 23104 | XaCDR, XaJJH, XaJqk, XaZcl, 22K, 229, 22A, 162[23], X76CG, XE1h4, XM01G, Xa7wI |
| Educational attainment | 6138, 10722 |  |
| MET | 22040 |  |
| Year of birth | 34 |  |
| Month of birth | 52 |  |
| Reported Sex | 31 |  |
| Smoking status | 20116 |  |
| Insulin use, cholesterol-lowering medication, blood pressure medication | 6177, 6153 |  |

^1^: Includes codes for height and weight which were used to compute BMI.

BMI: body mass index.

# **Table S4. Fields used to determine censoring dates**

| **Types of censoring date** | **UKB fields** | **Country code: Study termination date** | **City code: City name: Country code** |
| --- | --- | --- | --- |
| Lost-to-follow-up | *191.0.0* |  |  |
| Death | *40000.0.0* |  |  |
| Study termination | *0022.0.0, 40022.0.1, 40022.0.2*^1^ | PEDW: 2018-02-28  SMR: 2020-08-31  HES: 2020-09-30 |  |
|  | *54.0.0*^2^ | PEDW: 2018-02-28  SMR: 2020-08-31  HES: 2020-09-30 | 10003: Stockport (pilot): HES  11001: Manchester: HES  11002: Oxford: HES  11003: Cardiff: PEDW  11004: Glasgow: SMR  11005: Edinburgh: SMR  11006: Stoke: HES  11007: Reading: HES  11008: Bury: HES  11009: Newcastle: HES  11010: Leeds: HES  11011: Bristol: HES  11012: Barts: HES  11013: Nottingham: HES  11014: Sheffield: HES  11016: Liverpool: HES  11017: Middlesborough: HES  11018: Hounslow: HES  11020: Croydon: HES  11021: Birmingham: HES  11022: Swansea: PEDW  11023: Wrexham: PEDW |

^1^Contains country codes; ^2^Contains city codes. PEDW (Wales), SMR (Scotland), HES (England)

# **Table S5. Self-reported ethnicity categorization**

| **Recategorized self-reported ethnicity** | **UKB Field 21000 self-reported ethnicity** |
| --- | --- |
| Asian | “Any other Asian background”, “Chinese”, “Indian”, “Pakistani” |
| Black | “Any other Black background”, “Black or Black British”, “Caribbean” |
| Other | “Other ethnic group”, "White and Asian", "White and Black African", "White and Black Caribbean" |
| White | "British", “Irish” |

# **Table S6. Risk factors associated with the onset of cardiovascular disease, diabetic kidney disease, and diabetic retinopathy for Type 2 diabetes cohort of European ancestry**

|  | **CVD (1397/6681)** | | **DKD (1441/2075)** | | **DR (2357/2111)** | |
| --- | --- | --- | --- | --- | --- | --- |
| **Risk factor** | **HR (95% CI)** | ***P*** | **HR (95% CI)** | ***P*** | **HR (95% CI)** | ***P*** |
| **Age** | 1.06 (1.05-1.07) | <0.001 | 1.06 (1.05-1.07) | <0.001 | 1.02 (1.01-1.02) | <0.001 |
| **Sex** | 2.09 (1.83-2.38) | <0.001 | 0.88 (0.78-1.00) | 0.057 | 1.01 (0.93-1.10) | 0.788 |
| **ISCED** | 0.80 (0.72-0.89) | <0.001 |  |  |  |  |
| **Ever smoked** | 1.22 (1.09-1.36) | <0.001 | 1.16 (1.03-1.32) | 0.019 |  |  |
| **MET** |  |  | 0.94 (0.88-1.00) | 0.05 |  |  |
| **PRS2** |  |  |  |  | 1.04 (1.00-1.09) | 0.036 |
| **BMI** | 1.03 (1.02-1.04) | <0.001 | 1.03 (1.02-1.04) | <0.001 |  |  |
| **CCI** | 1.22 (1.18-1.28) | <0.001 | 1.14 (1.09-1.20) | <0.001 |  |  |
| **CV DBP** | 1.05 (1.00-1.11) | 0.048 | 0.93 (0.87-0.99) | 0.018 |  |  |
| **CV Trig.** | 1.07 (1.00-1.14) | 0.039 |  |  |  |  |
| **CV HDLc** | 1.11 (1.05-1.17) | <0.001 |  |  |  |  |
| **CV UACR** |  |  | 1.21 (1.10-1.33) | 0.001 |  |  |
| **Mean SBP** | 1.01 (1.01-1.02) | <0.001 | 1.01 (1.00-1.01) | <0.001 | 1.01 (1.00-1.01) | <0.001 |
| **Mean DBP** | 0.98 (0.97-0.99) | <0.001 |  |  | 0.99 (0.98-1.00) | 0.002 |
| **Mean Glucose** |  |  | 1.04 (1.02-1.06) | <0.001 | 1.05 (1.03-1.06) | <0.001 |
| **Mean Chol.** |  |  | 0.81 (0.75-0.87) | <0.001 | 0.84 (0.80-0.88) | <0.001 |
| **Mean Trig.** |  |  | 1.11 (1.04-1.18) | 0.003 | 1.05 (1.02-1.09) | 0.002 |
| **Mean HDLc** | 0.69 (0.56-0.84) | <0.001 |  |  |  |  |
| **Mean LDLc** | 1.13 (1.05-1.21) | <0.001 |  |  |  |  |
| **Mean eGFR** |  |  | 0.97 (0.97-0.98) | <0.001 |  |  |
| **Mean UACR** | 1.06 (1.02-1.09) | 0.003 | 1.29 (1.10-1.50) | 0.009 |  |  |

We employed the Cox proportional hazards model and a pooled step-wise variable selection procedure to simultaneously analyze imputed data and select important variables that are associated with major diabetes complications outcomes. Our base model included sex, and age, which were not subject to variable selection. Additionally, we included smoking status, BMI, self-reported medication status (insulin, blood pressure, and cholesterol drug), CCI, ISCED level, MET, polygenic risk scores (type 1 and type 2), and summary statistics of biomarker trajectories including average and CV of SBP, DBP, LDLc, HDLc, cholesterol, glucose, eGFR and urine ACR levels. The variables that were not selected do not appear in this table. The values of MET, average of urine ACR levels, CV of all biomarker levels, and type 1 and type 2 polygenic risk scores were standardized. ^1^number of cases and controls that were included in the model. CVD, cardiovascular disease; DKD, diabetic kidney disease; DR, diabetic retinopathy; ISCED, International Standard Classification of Education, dichotomous variable, 1 if ISCED level was greater than 2 and 0 otherwise; Smoked, dichotomous variable, 1 if a subject has ever smoked and 0 otherwise; MET, metabolic equivalents to resting state (hours/week); BMI, body mass index (kg/m^2^); CCI, Charlson Comorbidity Index; CV, coefficient of variation; Chol., total cholesterol (mmol/L); Trig., triglycerides (mmol/L); HDLc, high-density lipoprotein cholesterol (mmol/L); low-density lipoprotein cholesterol (mmol/dL); SBP, systolic blood pressure (mmHG); DBP, diastolic blood pressure (mmHG); Glucose (mmol/L); eGFR (mL/min/1.73m^2^); uACR, urine albumin to creatinine ratio (g/mmol).

# **Table S7. Selected variables using pooled step variable selection procedure with training data of Europeans with diabetes for each outcome**

| **Variables** | **CVD** | **DKD** | **DR** | **HS** | **IS** | **MI** | **PCI** | **ST** | **UA** |
| --- | --- | --- | --- | --- | --- | --- | --- | --- | --- |
| Age | 1 | 1 | 1 | 1 | 1 | 1 | 1 | 1 | 1 |
| Sex | 1 | 1 | 1 | 1 | 1 | 1 | 1 | 1 | 1 |
| ISCED | 1 |  |  |  |  | 1 |  | 1 | 1 |
| Ever smoked | 1 | 1 |  |  |  | 1 |  | 1 |  |
| MET |  |  |  |  |  |  | 1 |  |  |
| Insulin | 1 | 1 | 1 |  | 1 | 1 | 1 | 1 | 1 |
| BP Med. | 1 | 1 |  |  | 1 | 1 | 1 | 1 | 1 |
| Chol. Med. |  | 1 | 1 |  |  | 1 | 1 | 1 | 1 |
| BMI | 1 | 1 |  |  |  | 1 |  |  | 1 |
| CCI | 1 | 1 |  | 1 | 1 | 1 | 1 | 1 | 1 |
| CV SBP |  |  |  |  | 1 |  |  |  |  |
| CV DBP | 1 | 1 |  |  |  |  |  |  | 1 |
| CV Glucose |  |  | 1 |  |  |  |  |  |  |
| CV HDLc | 1 |  |  | 1 |  | 1 | 1 |  |  |
| CV UACR |  | 1 |  |  |  | 1 |  |  |  |
| Mean SBP | 1 | 1 | 1 |  |  | 1 | 1 |  |  |
| Mean DBP | 1 |  | 1 |  |  | 1 | 1 |  | 1 |
| Mean Glucose | 1 | 1 | 1 |  | 1 |  |  | 1 |  |
| Mean Chol. | 1 |  |  | 1 |  | 1 | 1 |  | 1 |
| Mean HDLc | 1 | 1 |  |  | 1 | 1 | 1 |  | 1 |
| Mean LDLc |  | 1 | 1 |  |  |  |  |  |  |
| Mean eGFR |  | 1 |  |  |  | 1 |  | 1 |  |
| Mean UACR | 1 | 1 |  |  | 1 | 1 |  |  |  |

“1” indicates a variable was selected.

# **Table S8. Selected variables using pooled step variable selection procedure with training data of Europeans with Type 2 diabetes for each outcome**

| **Risk factors** | **CVD** | **DKD** | **DR** | **HS** | **IS** | **MI** | **PCI** | **ST** | **UA** |
| --- | --- | --- | --- | --- | --- | --- | --- | --- | --- |
| Age | 1 | 1 | 1 | 1 | 1 | 1 | 1 | 1 | 1 |
| Sex | 1 | 1 | 1 | 1 | 1 | 1 | 1 | 1 | 1 |
| ISCED | 1 |  |  |  |  | 1 |  |  |  |
| Ever smoked | 1 | 1 |  |  |  | 1 |  |  |  |
| MET |  | 1 |  |  |  |  | 1 |  |  |
| Insulin | 1 | 1 | 1 |  |  |  |  |  |  |
| BP Med. | 1 | 1 |  |  | 1 | 1 | 1 | 1 | 1 |
| Chol. Med. |  | 1 | 1 |  |  | 1 | 1 |  | 1 |
| GRS1 |  |  |  |  | 1 |  |  |  |  |
| GRS2 |  |  | 1 |  |  |  | 1 |  |  |
| BMI | 1 | 1 |  |  |  | 1 |  |  |  |
| CCI | 1 | 1 |  | 1 | 1 | 1 | 1 | 1 | 1 |
| CV DBP |  | 1 |  |  |  | 1 |  |  |  |
| CV HDLc | 1 |  |  |  |  | 1 | 1 |  |  |
| CV UACR |  | 1 |  |  |  |  |  |  |  |
| Mean SBP | 1 | 1 | 1 |  |  | 1 | 1 |  |  |
| Mean DBP | 1 |  |  |  |  | 1 |  |  | 1 |
| Mean Glucose |  | 1 | 1 |  |  |  |  | 1 |  |
| Mean Chol. |  |  | 1 |  |  |  | 1 |  | 1 |
| Mean Trig. |  |  | 1 |  |  |  |  |  |  |
| Mean HDLc | 1 |  |  |  | 1 | 1 | 1 |  | 1 |
| Mean LDLc | 1 | 1 |  |  |  | 1 |  |  |  |
| Mean eGFR |  | 1 |  |  |  |  |  |  |  |
| Mean UACR | 1 | 1 |  |  | 1 |  |  | 1 |  |

“1” indicates a variable was selected.

| 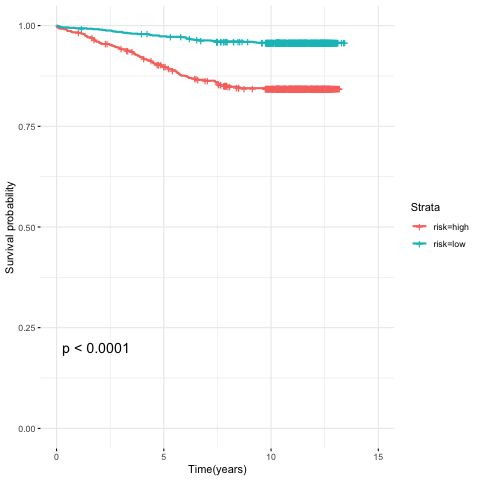a  | b  | c  |
| --- | --- | --- |
| d  | e  | f  |
| g  | h  | i  |

# **Figure S1. Kaplan-Meier curves comparing survival probability of experiencing major diabetes complication outcomes between low- and high-risk groups**

Individuals were assigned to high-risk group if their risk scores were greater than the median risk score and to low-risk group if otherwise. **a.** CVD (cardiovascular disease), **b.** DKD (diabetic kidney disease), **c.** DR (diabetic retinopathy), **d.** Myocardial Infarction (MI), **e.** Unstable Angina (UA), **f.**, Ischemic Stroke (IS), **g.** Hemorrhagic Stroke (HS), **h.** any Stroke (ST), **i.** Percutaneous Coronary Intervention (PCI

# **Figure S2. Comparison of prediction performance of risk scores as measured by AUC for Type 2 diabetes subjects**

“n.case/n.control” refers to the number of cases and controls included in the validation data. CVD (cardiovascular disease); DKD (diabetic kidney disease); DR (diabetic retinopathy); HS (hemorrhagic stroke); IS (ischemic stroke); MI (myocardial infarction); PCI (percutaneous coronary intervention); ST (stroke); UA (unstable angina)

# **Figure S3. Comparison of prediction performance of risk scores as measured by AUC for non-European diabetes subjects**

“n.case/n.control” refers to the number of cases and controls included in the validation data. CVD (cardiovascular disease); DKD (diabetic kidney disease); DR (diabetic retinopathy); HS (hemorrhagic stroke); IS (ischemic stroke); MI (myocardial infarction); PCI (percutaneous coronary intervention); ST (stroke); UA (unstable angina)
